# Banxia Xiexin tang for gastro-oesophageal reflux disease: A protocol for a systematic review of controlled trials

**DOI:** 10.1097/MD.0000000000010393

**Published:** 2018-04-27

**Authors:** Bohyung Kang, Haesol Lee, Youkyung Choi, Chanyong Jeon, Ju Ah Lee

**Affiliations:** Department of Korean Internal Medicine, College of Korean Medicine, Gachon University.

**Keywords:** Banxiaxiexin-Tang, gastro-oesophageal reflux disease, protocol, systematic review

## Abstract

**Background:**

Gastrooesophageal reflux disease (GORD) is one of the most common gastrointestinal diseases encountered in clinical practice. The aim of the present study is thus to systematically review the literature, including Asian studies, to assess the efficacy and safety of Banxia Xiexin tang (BXT) for the treatment of GORD.

**Methods and analysis:**

Eleven databases will be searched for studies conducted through March 2018. We will include randomized controlled trials (RCTs) of BXT as a treatment for GORD. All RCTs on BXT or related formulations will be included. The risk of bias will be assessed using the Cochrane Risk of Bias Assessment Tool, while confidence in the cumulative evidence will be evaluated using the Grading of Recommendations, Assessment, Development and Evaluation (GRADE) tool.

**Ethics and dissemination:**

This systematic review will be published in a peer-reviewed journal and will also be disseminated electronically and in print. The review will be updated to inform and guide healthcare practices.

**Registration number:**

CRD42018087056

## Abstract 2

Supplemental Digital Content is available in the text

## 1 Introduction

Gastro-oesophageal reflux disease (GORD) is one of the most common gastrointestinal diseases encountered in clinical practice.^[1]^ GORD is a disease in which the contents of the stomach reflux into the oesophagus and cause uncomfortable symptoms or complications. Uncomfortable symptoms of gastro-oesophageal reflux may have a negative impact on the quality of life, and complications include oesophagitis, as well as asthma, aspiration pneumonia, and laryngitis caused by reflux. The major symptoms are heartburn and acid regurgitation. These symptoms are often accompanied by chest pain, chronic cough, hoarseness, and asthma, as well as a strange sensation in the neck, known to be caused by a lower level of relaxation and lower pressure of the oesophageal sphincter.^[2]^

Treatment of GORD includes changes in the lifestyle, drug therapy such as the use of H2 receptor antagonists to suppress gastric acid secretion or proton pump inhibitors, and surgical therapy. In some patients, only changes in the lifestyle may improve the symptoms, but in most cases, inhibition of gastric acid secretion is required. After discontinuation of the medication, the recurrence rate is high.^[3]^ For this reason, other methods of treatment of GORD have been introduced.^[4]^ In particular, interest in the use of complementary and alternative medicine (CAM) for treating GORD has been increasing worldwide. So far, there have been several reports on the therapeutic effects of traditional East Asian medicines, such as Ijintanggamibang,^[5]^*Lonicera japonica* extract,^[6]^ and *Evodia officinalis* Dode extract,^[7]^ which are experimentally used in Korea. In Japan, studies have been conducted on recurrent gastro-oesophageal reflux disease, which suggested the use of Yukgunja-tang.^[8]^ In China, studies are being conducted to compare treatment rates between traditional East Asian medicines and conventional medicines, mainly through patient-control studies.^[9]^

However, despite the frequent clinical use of Banxia Xiexin tang (BXT) for GORD, there are few systematic reviews of its effectiveness. Therefore, this study will seek to systematically review randomized controlled trials (RCTs) to assess the effectiveness and safety of BXT for the treatment of GORD. This protocol will describe search methods involving Asian databases and will therefore permit the inclusion of the CAM-related literature, which cannot be retrieved from English-language databases.

## 2 Methods

### 2.1 Study registration

This study will follow the guidelines outlined in the Preferred Reporting Items for Systematic Reviews and Meta-Analysis (PRISMA) statement for meta-analyses of healthcare interventions;^[10]^ additionally, the current protocol adheres to the PRISMA Protocols (PRISMA-P).^[11]^ The protocol for this systematic review has been registered on PROSPERO 2018 under the number CRD42018087056.

### 2.2 Ethic approval

This study is the protocol of the systematic review. Therefore, the approval of the Institutional Review Board was not needed.

### 2.3 Data sources

The following databases will be searched from inception to the present date: MEDLINE, EMBASE, the Cochrane Central Register of Controlled Trials (CENTRAL), AMED, and CINAHL. We will also search 6 Korean medical databases (i.e., OASIS, the Korean Traditional Knowledge Portal, the Korean Studies Information Service System, KoreaMed, the Korean Medical Database, and DBPIA) and 3 Chinese databases, including CNKI (i.e., the China Academic Journals, the China Doctoral Dissertations, and Master's Theses Full-Text Database, the China Proceedings of Conference Full-Text Database, and the Century Journal Project), Wanfang and VIP. In addition, we will search a Japanese database and conduct nonelectronic searches of conference proceedings and our own article files. The search strategy that will be applied to the MEDLINE database is presented in Supplement 1. Similar search strategies will be used for the other databases.

## 3 Types of studies

Prospective randomized controlled trials (RCTs) that include BXT as the sole treatment or as an adjunct to other treatments and that provide the same treatment to the control and intervention groups will be included. Trials comparing acupuncture with any type of control intervention will also be included. No language restrictions will be imposed.

Hard copies of all articles will be obtained and read in full.

## 4 Types of participants

The participants will include patients who were diagnosed with gastro-oesophageal reflux disease (GORD). Participants who have both GORD and accompanying diseases will be excluded. There will be no restrictions based on other conditions, such as the age, sex, or symptom severity.

## 5 Types of interventions

Studies that evaluate any type of intervention will be included. Interventions using any type of formulations (i.e., decoction, tablet, pill, powder, and/or nasal spray) of BXT will be eligible for inclusion. The compositions of the treatments will be reviewed, and interventions involving herbal combinations that differ from the original BXT from the perspective of traditional East Asian medicine will be excluded from this review.

## 6 Data extraction

Hard copies of all articles will be obtained and read in full. Two authors (BK and HL) will perform the data extraction and quality assessment using a predefined data extraction form. In addition, all interventions applying acupuncture will be extracted using the Standards for Reporting Interventions in Clinical Trials of Acupuncture (STRICTA).

The risk of bias will be assessed using the Cochrane Handbook Risk of Bias Assessment Tool version 5.1.0, which considers random sequence generation, allocation concealment, blinding of participants and personnel, blinding of outcome assessment, incomplete outcome data, selective reporting, and other sources of bias.^[12]^ The results of the assessments will be presented using scores of “L,” “U,” and “H,” with “L” indicating a low risk of bias, “U” indicating an uncertain risk of bias, and “H” indicating a high risk of bias. Disagreements will be resolved by discussion between all authors. When disagreements regarding selection cannot be resolved through discussion, an arbiter (JAL) will make the final decision.

## 7 Data collection and synthesis

### 7.1 Outcome measures

#### 7.1.1 Primary outcomes

The primary outcomes will be the therapeutic effects of treatment with BXT.

#### 7.1.2 Secondary outcomes

The secondary outcomes will include the safety, based on adverse effects. In addition, improvement of symptoms (acid flow, chest pain, difficulty swallowing, lower back pain, nausea, and feeling of foreign bodies in the throat) will be included as the secondary outcomes.

### 7.2 Assessment of bias in the included studies

The risk of bias will be assessed using the Cochrane Handbook Risk of Bias Assessment Tool version 5.1.0, which considers random sequence generation, allocation concealment, blinding of participants and personnel, blinding of outcome assessment, incomplete outcome data, selective reporting, and other sources of bias.^[12]^

We will independently assess the bias of the included studies according to the criteria in the Cochrane Handbook, version 5.1.0. These criteria include random sequence generation, allocation concealment, blinding of participants and personnel, blinding of outcome assessment, incomplete outcome data, selective reporting, and other sources of bias.^[12]^

The results of the assessments will be presented using scores of “L,” “U,” and “H,” with “L” indicating a low risk of bias, “U” indicating an uncertain risk of bias, and “H” indicating a high risk of bias. Disagreements will be resolved by discussion between all authors.

### 7.3 Data synthesis

Differences between the intervention and control groups will be assessed. Mean differences (MDs) with 95% confidence intervals (CIs) will be used to measure the effects of treatment for continuous data. We will convert other forms of data into MDs. For outcome variables on different scales, we will use standard MDs (SMDs) with 95% CIs. For dichotomous data, we will present treatment effects as relative risks (RRs) with 95% CIs; other binary data will be converted into RR values.

All statistical analyses will be conducted using the Cochrane Collaboration's software programme Review Manager (RevMan) version 5.3. (Copenhagen, The Nordic Cochrane Centre, the Cochrane Collaboration, 2012) for Windows. We will contact the corresponding authors of studies with missing information to acquire and verify the data whenever possible. When appropriate, we will pool the data across studies to conduct a meta-analysis using fixed or random effects. We will use the GRADE Pro software from Cochrane Systematic Reviews to create a Summary of Findings table.

### 7.4 Unit of analysis issues

For crossover trials, data from the first treatment period will be used. For trials that assessed more than one control group, the primary analysis will combine data from each control group. Subgroup analyses of the control groups will be performed. Each patient will be counted only once in the analyses.

### 7.5 Addressing missing data

Intention-to-treat analyses, including all randomized patients, will be performed. For patients with missing outcome data, the last observation carry-forward analysis will be performed. When individual patient data are initially unavailable, we will review the original source or the published trial reports for these data.

### 7.6 Assessment of heterogeneity

Based on the data analysis, we will use random- or fixed-effect models to conduct the meta-analysis. Chi-squared and *I*-squared tests will be used to evaluate the heterogeneity of the included studies. *I*^2^ values of >50 will indicate high heterogeneity. When heterogeneity is observed, subgroup analyses will be conducted to explore possible causes.^[13]^

### 7.7 Assessment of reporting biases

Funnel plots will be generated to detect reporting biases when a sufficient number of included studies (at least 10 trials) is available.^[14]^ However, since funnel plot asymmetries are not equivalent to publication biases, we will aim to determine the possible reasons for any asymmetries in the included studies, such as small study size effects, poor methodological quality and true heterogeneity.^[14,15]^

## 8 Discussion

This systematic review will provide a summary of the current evidence related to the effectiveness of BXT in the treatment of symptoms of gastro-oesophageal reflux disease (GORD). BXT was first introduced in SangHanLoan Medical Books, a medical text written by Jang Jung Kyung in China.^[16]^ Among alternative approaches, BXT is a widely used remedy for gastrointestinal discomfort in Asian countries, including China, Japan, and Korea.^[17]^

BXT is an important formula for treating fullness with accumulation of intermingled cold and heat due to spleen and stomach asthenia. Among the ingredients of BXT, *Radix Scutellariae* Baicalensis and *Rhiz*o*m*a *Copidis* clear heat; *Rhizom*a *Pinelliae* and *Rhizoma Zingiberis* dispel accumulation and cold; *Radix Ginseng*, *Radix Glycyrrhizae* and *Fructus Jujubae* benefit Qi and cure asthenia. This formula includes both cold and warm drugs and bitter and acrid drugs with descending and expelling effects to invigorate Qi.^[17]^

The results of modern pharmacological research have suggested that BXT has effects on a variety of diseases. BXT has effects on promoting gastrointestinal peristalsis, preventing reflux, protecting the gastric mucosa, invigorating the immune system, and improving the body's ability to resist hypoxia. BXT was mainly used to treat illnesses of the digestive system, such as reflux oesophagitis, acute and chronic gastritis, peptic ulcer, dyspepsia, and constipation.^[18]^

Currently, no systematic reviews of the effects of BXT on GORD have been published. Although many systematic reviews have been conducted on this herbal medicine, important information on its usage was not extracted and has been missing. All types of BXT will be included in this study. In particular, we will consider special features of interventions in full reviews of BXT for GORD.

## Author contributions

BK, CJ, YC, and HL conceived the study, developed the criteria, searched the literature, analysed the data, wrote the protocol, and also conducted a preliminary search. JAL assisted in searching the Chinese literature and extracting the data and revised the manuscript. All authors have read and approved the final manuscript.

**Conceptualization:** BoHyung Kang, Ju Ah Lee.

**Funding acquisition:** Ju Ah Lee.

**Investigation:** Ju Ah Lee.

**Methodology:** Ju Ah Lee.

**Validation:** Chanyong Jeon.

**Writing – original draft:** BoHyung Kang.

**Writing – review & editing:** Haesol Lee, Youkyung Choi, Chanyong Jeon, Ju Ah Lee.

## Supplementary Material

Supplemental Digital Content
