# Supplementary material for: Banxia Xiexin tang for gastro-oesophageal reflux disease: A protocol for a systematic review of controlled trials
Source: Medicine (Baltimore). 2018 Apr 27;97(17):e0393. doi: 10.1097/MD.0000000000010393 (PMC5944504; doi:10.1097/MD.0000000000010393)
Supplement: Supplemental Digital Content [file medi-97-e0393-s001.docx]

**Supplement 1. Search strategy for the MEDLINE database**

#1 Search "GERD"[Mesh]

#2 Search "GERD"[Title/Abstract]

#3 Search ('gastroesophageal reflux disease'[Title/Abstract] OR 'reflux esophagitis'[Title/Abstract])

#4 Search ("Barrett’s esophagus"[Title/Abstract])

#5 #1 OR #2 OR #3 OR #4

#6 Search "Banxiaxiexin"[Mesh]

#7 Search "Banxiaxiexintang"[Title/Abstract]

#8 Search “Banxiaxiexin-tang"[Title/Abstract]

#9 半夏瀉心湯 [Title/Abstract]

#10 "Banxiaxiexin Decoction"[Title/Abstract]

#11 Search (#6 OR #7 OR #8 OR #9 OR #10)

#12 #5 AND #11
